# Do 21-Gene Recurrence Score Influence Chemotherapy Decisions in T1bN0 Breast Cancer Patients?

**DOI:** 10.3389/fonc.2020.00708

**Published:** 2020-05-12

**Authors:** Jing Yu, Jiayi Wu, Ou Huang, Jianrong He, Zhu Li, Weiguo Chen, Yafen Li, Xiaosong Chen, Kunwei Shen

**Affiliations:** Department of General Surgery, Comprehensive Breast Health Center, Ruijin Hospital, Shanghai Jiao Tong University School of Medicine, Shanghai, China

**Keywords:** breast cancer, 21-gene recurrence score, T1bN0 tumors, chemotherapy decisions, treatment adherence

## Abstract

**Purpose:** Hormone receptor (HR)-positive breast cancer patients with tumor size ≤1.0 cm and negative node have favorable outcomes. The 21-gene Recurrence Score (RS) could predict response to chemotherapy for HR+ breast cancer, but its role in T1bN0 disease is challenging.

**Methods:** T1bN0 breast cancer patients diagnosed between January 2014 and June 2019 with RS results were included and categorized as Low- (RS < 18), Intermediate- (RS 18–30), or High-risk (RS > 30) groups. Univariate and multivariate analysis were used to assess factors associated with RS distribution and chemotherapy recommendation. Chemotherapy decisions change and patient adherence after 21-gene RS testing were also evaluated.

**Results:** Among 237 patients with T1bN0 tumors, proportions of Low-, Intermediate-, and High-risk RS were 19.8, 63.3, and 16.9%, respectively. Multivariate analysis found that ER expression (*P* = 0.011), PR expression (*P* < 0.001), and Ki-67 index (*P* = 0.001) were independently associated with RS distribution. Adjuvant chemotherapy was recommended for 31.6% of patients, which was more frequently given to patients with higher tumor grade [Odds ratio (OR) = 2.99 for grade II, OR = 59.19 for grade III, *P* = 0.006], lymph vascular invasion (OR = 8.22, *P* = 0.032), Luminal-B subtype (OR = 5.68, *P* < 0.001), and Intermediate-to High-risk RS (OR = 10.01 for Intermediate-risk, OR = 192.42 for High-risk, *P* < 0.001). Chemotherapy decision change was found in 18.6% of patients, mainly in those with Intermediate- to High-risk RS tumor with the majority from no-chemotherapy to chemotherapy. The treatment compliance rate after the 21-gene RS testing with MDT was 95.4%.

**Conclusion:** RS category was related to ER, PR, and Ki-67 expression, which was recognized as an independent factor of chemotherapy recommendation in T1bN0 breast cancer. The 21-gene RS testing would lead to a chemotherapy decision change rate of 18.6% as well as a high treatment adherence, which can be applied in T1bN0 patients.

## Introduction

Breast cancer, the most common cancer diagnosed in women, is characterized by molecularly heterogeneous. The molecular subtype based on Estrogen receptor (ER), Progesterone receptor (PR), Human epidermal growth factor receptor-2 (HER2), and Ki-67 index could predict prognosis as well as response to treatment. The hormone receptor (HR)-positive breast cancer accounts for 60–75% of all cases (1, 2), which always shows a favorable prognosis with adjuvant endocrine therapy and might avoid the cytotoxicity of chemotherapy.

In the past generation, several gene-based assays shed light on the genetic feature of breast cancer, among which the 21-gene RS testing is a reliable and widely used one (3). The 21-gene assay, presenting as a numerical variable ranging from 0 to 100 after calculating by a specific algorithm (4), is carried out by reverse transcription-PCR (RT-PCR) on fixed, paraffin-embedded tumor tissues with a panel composing of 16 cancer-related genes and 5 reference genes. The original study categorized RS < 18 as Low-risk, RS 18–30 as Intermediate-risk, and RS > 30 as High-risk, in order to predict the distant recurrence rate of HR-positive, lymph-node negative patients treated with tamoxifen (4). Then, the treatment benefit of chemotherapy was certified in patients with High-risk RS in the NSABP B-20 cohort (5). The pivotal study TAILORx demonstrated that patients with RS < 25 derive a little survival benefit from adjuvant chemotherapy, especially for the elderly (6).

With the prevalence of common screening of breast, including clinical breast exams, mammograms, and ultrasounds, the incidence of small breast cancer has increased in the past few decades (7). Those patients always harbor promising clinical outcomes with 5-year disease-free survival (DFS) higher than 90% (8–10). This may give us a hint that small tumors share different biological features and may deserve treatment de-escalation. Under this circumstance, choosing what kind of biomarkers or tools to guide therapeutic decision-making is well worth discussing. Notably, the TAILORx trial included tumors >1.0 cm or tumors between 0.5 and 1.0 cm with intermediate and/or high grade (11).

To our known, there were several studies focus on the survival outcome of patients with small tumors. However, the usage and influence of RS on small tumors were rarely researched, especially in tumors that sized from 0.5 to 1.0 cm (T1b). In the current study, we aim to evaluate the RS distribution, adjuvant chemotherapy decision, and therapeutic recommendation change due to the 21-gene RS testing in patients with T1b, HR-positive/HER2-negative lymph node-negative breast cancer.

## Materials and Methods

### Study Population and Information

Clinical data including clinicopathological patterns, treatment decision and follow up information were extracted from Shanghai Jiao Tong University Breast Cancer Database (SJTU-BCDB). All patients were diagnosed between January 2014 and June 2019, at Ruijin Hospital, Shanghai Jiao Tong University School of Medicine, Shanghai, China. Patients were eligible if they met the following criteria: (1) HR-positive and HER2-negative, primary invasive breast cancer, (2) underwent the 21-gene RS testing, (3) the longest diameter of tumor was larger than 0.5 cm and no more than 1.0 cm. Exclusion criteria included: (1) patients with multifocal or multicenter tumor, (2) tumor was larger than 1.0 cm, (3) patients who had malignant breast lesions other than HR-positive and HER2-negative tumor, (4) male breast cancer, (5) pathologically confirmed lymph node-positive. The study was approved by the Ethical Committees of Ruijin Hospital, Shanghai Jiao Tong University School of Medicine.

### Pathological and Immunohistochemical (IHC) Analysis

Pathological diagnosis was performed by the Department of pathology, Ruijin Hospital. IHC studies of ER, PR, HER2, and Ki-67 were performed on formalin-fixed, paraffin-embedded tissue sections, using antibodies as follow: ER: clone 1D5 (rabbit monoclonal, Gene), PR: clone PR636 (mouse monoclonal, Dako), HER2: 4B5 (rabbit monoclonal, Roche), Ki67: MIB-1 (mouse monoclonal, Dako). Hormone receptor including ER and PR were considered positive if nuclear staining in ≥1% of tumor cells. 0 to 1+ by IHC or negative on FISH was recognized as HER2 negativity. Ki67 index was characterized as the proportion of positive nuclear staining cells among ≥1,000 invasive tumor cells. The cut-off values for ER expression was 50% (12), which was 20% for PR status (13) and 14% for Ki-67 index (13). Luminal A-like was identified if IHC shows ER positive, PR ≥20% and Ki-67 <14%, and Luminal B-like was defined as ER positive, and PR <20% or Ki-67 ≥14% (13).

### The 21-Gene RS Assay Testing

The 21-gene RS assay was performed on formalin-fixed, paraffin-embedded tissue. RNA was extracted from two 10 μm unstained sections on hematoxylin and eosin-stained slides and was measured after ensuring the absence of DNA contamination. Gene-specific reverse transcription was performed followed by standardized quantitative RT-PCR reactions in 96 well plates using Applied Biosystems (Foster City, CA) 7500 Real-Time PCR System. The expression level of each cancer-related gene was normalized by 5 reference genes, and the 21-gene recurrence score was then calculated by a specific algorithm. RS was stratified as categorical variables with standard cutoffs (RS < 18 as Low-risk, RS 18–30 as Intermediate-risk, and RS > 30 as High-risk) (4) and TAILORx cutoffs (RS < 11 as Low-risk, RS 11–25 as Intermediate-risk, and RS > 25 as High-risk) (6). The following text used the standard cutoffs in general unless otherwise noted.

### Treatment Decision and Actual Usage of Chemotherapy

The Multidisciplinary Team (MDT), consisting of breast surgeons, oncologists, radiologists, and specialized breast nurses, would make the first-round MDT treatment decision after knowing the clinicopathological parameters of the surgical lesion but without 21-gene RS result (Pre-RS decision). And Post-RS decision would be determined after the results of the 21-gene testing were presented, which will also be recorded as the final recommendation in the second-round MDT. The actual administration of the chemotherapy was confirmed by the follow-up information.

### Statistical Analysis

Chi-square test (exact Fisher test if necessary) was used to evaluate the RS distribution and chemotherapy usage among patients with different clinicopathological characteristics. Multiple logistic regression models were used to generate adjusted odds ratios (ORs) with 95% confidence intervals (CIs) in order to assess factors associated with RS distribution and chemotherapy. Two-sided *p* < 0.05 were required for statistical significance. All statistical analyses were carried out by SPSS version 25.0.

## Results

### Baseline Clinicopathological Characteristics

A total of 253 patients were reviewed and 237 were included. Sixteen patients were excluded, including 12 patients who had multiple focal and the largest tumor >1 cm, one patient had triple-negative breast cancer, one patient had lymph-node metastasis and two male patients. Patients categorized as having Low- (<18), Intermediate- (18–30), and High-risk (>30) RS were 47 (19.8%), 150 (63.3%), and 40 (16.9%), respectively. The median age was 54.30 ± 10.94 years old and 148 patients (62.4%) were elder than 50 years of age. There were 79 (33.3%) patients had comorbidity, and 130 patients (54.9%) were post-menopausal. Grade I, II, and III tumors accounted for 26.6, 56.1, and 4.2%. Only 30 patients (12.7%) had histologic type other than invasive ductal carcinoma (IDC), and 9 patients (3.8%) had lymph vascular invasion. There were 16 (6.8%) patients with PR negative tumor, among which one patient had RS < 18, 11 patients had an RS of 18–30, and 4 patients had RS > 30. The proportion of patients with ER ≥50%, PR ≥20%, and Ki-67 ≥14% was 97.0, 79.7, and 35.4%, respectively. There were 119 patients (50.2%) had Luminal-B like breast cancer.

### Clinicopathological Characteristics According to RS Groups

In univariate analysis, ER status (*P* = 0.011), PR status (*P* = 0.002), Ki-67 index (*P* = 0.001), and Luminal subtype (*P* < 0.001) were significantly associated with categorical RS distribution (Table 1). Proportions of patients with PR < 20% were 4.3, 21.3, and 35.0% in the Low-, Intermediate-, and High-risk groups. And 25.5, 32.0, and 60.0% patients had Ki-67 ≥14% in these groups, respectively. Luminal-B like tumors accounted for 31.9, 48.0, and 80.0% in three categorical RS groups.

**Table 1:** Clinicopathologic characteristics of T1bN0 patients according to Recurrence Score.

	**Total (*n* = 237)**	**RS < 18 (*n* = 47)**	**RS 18–30 (*n* = 150)**	**RS ≥ 31 (*n* = 40)**	***P*-value**
Age (years)					0.657
≤ 50	89 (37.6%)	15 (31.9%)	59 (39.3%)	15 (37.5%)	
>50	148 (62.4%)	32 (68.1%)	91 (60.7%)	25 (62.5%)	
Comorbidity					0.484
No	158 (66.7%)	30 (63.8%)	104 (69.3%)	24 (60.0%)	
Yes	79 (33.3%)	17 (36.2%)	46 (30.7%)	16 (40.0%)	
Menstrual status					0.107
Pre-menopausal	107 (45.1%)	23 (48.9%)	72 (48.0%)	12 (30.0%)	
Post-menopausal	130 (54.9%)	24 (51.1%)	78 (52.0%)	28 (70.0%)	
Histologic type					0.873
IDC	207 (87.3%)	40 (85.1%)	132 (88.0%)	35 (87.5%)	
Non-IDC	30 (12.7%)	7 (14.9%)	18 (12.0%)	5 (12.5%)	
Tumor Grade					0.481
Grade I	63 (26.6%)	15 (31.9%)	40 (26.7%)	8 (20.0%)	
Grade II	133 (56.1%)	24 (51.1%)	86 (57.3%)	23 (57.5%)	
Grade III	10 (4.2%)	1 (2.1%)	5 (3.3%)	4 (10.0%)	
Unknown	31 (13.1%)	7 (14.9%)	19 (12.7%)	5 (12.5%)	
LVI					1.000^*^
Yes	9 (3.8%)	2 (4.3%)	6 (4.0%)	1 (2.5%)	
No	228 (96.2%)	45 (95.7%)	145 (96.0%)	39 (97.5%)	
ER status					0.011^*^
<50%	7 (3.0%)	4 (8.5%)	1 (0.7%)	2 (5.0%)	
≥50%	230 (97.0%)	43 (91.5%)	149 (99.3%)	38 (95.0%)	
PR status					0.002
<20%	48 (20.3%)	2 (4.3%)	32 (21.3%)	14 (35.0%)	
≥20%	189 (79.7%)	45 (95.7%)	118 (78.7%)	26 (65.0%)	
Ki-67 index					0.001
<14%	153 (64.6%)	35 (74.5%)	102 (68.0%)	16 (40.0%)	
≥14%	84 (35.4%)	12 (25.5%)	48 (32.0%)	24 (60.0%)	
Luminal subtype					<0.001
Luminal A-like	118 (49.8%)	32 (68.1%)	78 (52.0%)	8 (20.0%)	
Luminal B-like	119 (50.2%)	15 (31.9%)	72 (48.0%)	32 (80.0%)	

*RS, Recurrence Score; IDC, invasive ductal carcinoma; LVI, Lymph vascular invasion; ER, Estrogen receptor; PR, Progesterone receptor*.

* *Fisher's exact test*.

Multivariate analysis demonstrated that ER status (*P* = 0.011), PR status (*P* < 0.001), and Ki-67 index (*P* = 0.001) remained independent factors of RS distribution while Luminal subtype was no longer significant (Table 2). Compared with patients with RS < 18, patients with RS 18–30 (OR = 7.28, 95% CI 1.58–33.56, *P* = 0.011) or RS > 30 (OR = 15.48, 95% CI 3.07–78.17, *P* = 0.001) had lower PR expression. Meanwhile, patients with RS 18–30 were less likely to had ER expressed <50% (OR = 0.06, 95% CI 0.01–0.58, *P* = 0.016), and patient with RS > 30 had higher Ki-67 index (OR = 5.00, 95% CI 1.93–12.97, *P* = 0.001).

**Table 2:** Multivariant analysis of characteristics associated with Recurrence score in T1bN0 patients.

	**RS 18–30 (*****n*** **=** **150)**			**RS** **>** **30 (*****n*** **=** **40)**			***P-*value**
	**OR**	**95% CI**	***P-*value**	**OR**	**95% CI**	***P-*value**	
ER status							0.011
<50%	0.06	0.01–0.58	0.016	0.56	0.07–4.24	0.571	
≥50%	1			1			
PR status							<0.001
<20%	7.28	1.58–33.56	0.011	15.48	3.07–78.17	0.001	
≥20%	1			1			
Ki-67 index							0.001
<14%	1			1			
≥14%	1.37	0.64–2.94	0.412	5.00	1.93–12.97	0.001	
Luminal subtype							0.543
Luminal-A	1			1			
Luminal-B	0.46	0.06–3.84	0.473	0.95	0.08–10.95	0.965	

*RS, Recurrence score; OR, Odds ratio; CI, Confidence interval; ER, Estrogen receptor; PR, Progesterone receptor*.

We further evaluate the clinicopathological characteristic of patients among different RS categories according to the TAILORx trial definition (RS < 11 as Low-risk, RS 11–25 as Intermediate-risk, and RS > 25 as High-risk). There were only four patients who had RS < 11, so we combined the RS < 11 group and RS 11–25 group together. Univariate analysis demonstrated that PR < 20% (*P* = 0.003), Ki-67 ≥14% (*P* = 0.006), and Luminal-B like tumor (*P* < 0.001) were related to High-risk RS. Menstrual status was also marginally associated with RS distribution (Table S1). In multivariate analysis, only Pre-menopausal (OR = 1.94, 95% CI 1.02–3.68, *P* = 0.043) and Luminal-B like tumor (OR = 3.81, 95% CI 1.06–13.71, *P* = 0.041) were significantly related to High-risk RS (Table S2).

### Adjuvant Chemotherapy Decision in T1bN0 Patients

Adjuvant chemotherapy was recommended for 75 out of 237 (31.6%) patients. Univariate analysis demonstrated that age ≤50 years (40.4 vs. 26.4%, *P* = 0.024), higher tumor grade (90.0, 37.6, and 14.3% for grade III, II, I, *P* < 0.001), lymph vascular invasion (66.7 vs. 30.3%, *P* = 0.030), PR < 20% (54.2 vs. 25.9%, *P* < 0.001), Ki-67 ≥14% (56.0 vs. 18.3%, *P* < 0.001), and Luminal-B like tumor (52.1 vs. 11.0%, *P* < 0.001) were significantly associated with chemotherapy recommendation. Compared with RS < 18 group (2 out of 47, 4.3%), RS 18–30 group (39 out of 150, 26.0%) and RS >30 group (34 out of 40, 85.0%) were more likely to recommend for adjuvant chemotherapy (*P* < 0.001, Table 3).

**Table 3:** Factors associated with chemotherapy decision in T1bN0 patients.

	**Chemo (*n* = 75)**	**No-chemo (*n* = 162)**	***P*-value**
Age (years)			0.024
≤ 50 y	36 (40.4%)	53 (59.6%)	
>50 y	39 (26.4%)	109 (73.6%)	
Comorbidity			0.236
No	54 (34.2%)	104 (65.8%)	
Yes	21 (26.6%)	58 (73.4%)	
Menstrual status			0.149
Pre-menopausal	39 (36.4%)	68 (63.6%)	
Post-menopausal	36 (27.7%)	94 (72.3%)	
Histologic type			0.142
IDC	69 (33.3%)	138 (66.7%)	
Non-IDC	6 (20.0%)	24 (80.0%)	
Tumor Grade			<0.001
Grade I	9 (14.3%)	54 (85.7%)	
Grade II	50 (37.6%)	83 (62.4%)	
Grade III	9 (90.0%)	1 (10.0%)	
Unknown	7 (22.6%)	24 (77.4%)	
LVI			0.030^*^
Yes	6 (66.7%)	3 (33.3%)	
No	69 (30.3%)	159 (69.7%)	
ER status			1.000^*^
<50%	2 (2.7%)	5 (3.1%)	
≥50%	73 (97.3%)	157 (96.9%)	
PR status			<0.001
<20%	26 (54.2%)	22 (45.8%)	
≥20%	49 (25.9%)	140 (74.1%)	
Ki-67 index			<0.001
<14%	28 (18.3%)	125 (81.7%)	
≥14%	47 (56.0%)	37 (44.0%)	
Luminal subtype			<0.001
Luminal A-like	13 (11.0%)	105 (89.0%)	
Luminal B-like	62 (52.1%)	57 (47.9%)	
Recurrence Score			<0.001
RS <18	2 (4.3%)	45 (95.7%)	
RS 18–30	39 (26.0%)	111 (74.0%)	
RS > 30	34 (85.0%)	6 (15.0%)	

*RS, Recurrence score; IDC, Invasive ductal carcinoma; LVI, Lymph vascular invasion; ER, Estrogen receptor; PR, Progesterone receptor; Chemo, Chemotherapy; No-chemo, No-chemotherapy*.

* *Fisher's exact test*.

In multivariate analysis, higher tumor grade (OR = 2.99, 95% CI 1.07–8.33, *P* = 0.036 for grade II; OR = 59.19, 95% CI 4.22–829.43, *P* = 0.002 for grade III; *P* = 0.006), lymph vascular invasion (OR, 8.22, 95% CI 1.19–56.69, *P* = 0.032), Luminal-B like tumor (OR = 5.68, 95% CI 2.45–13.17, *P* < 0.001), and RS (*P* < 0.001) were significantly associated with chemotherapy recommendation (Table 4). The chemotherapy decision was more likely to be prescribed on Intermediate-risk RS patients (OR = 10.01, 95% CI 1.82–54.99, *P* = 0.008) and High-risk RS group (OR = 192.42, 95% CI 26.87–1377.73, *P* < 0.001).

**Table 4:** Multivariant analysis of characteristics associated with chemotherapy decision in T1bN0 patients.

	**OR**	**95% CI**	***p*-value**
Age (≤ 50 vs. 50 y)	2.20	0.99–4.88	0.052
Tumor Grade			0.006
Grade II vs. Grade I	2.99	1.07–8.33	0.036
Grade III vs. Grade I	59.19	4.22–829.43	0.002
Unknown vs. Grade I	1.06	0.23–4.92	0.943
LVI (Positive vs. negative)	8.22	1.19–56.69	0.032
PR status (Negative vs. positive)	2.36	0.56–10.02	0.245
Ki-67 index (≥14 vs. <14%)	1.37	0.31–6.12	0.679
Luminal subtype (Luminal-B vs. Luminal A like)	5.68	2.45–13.17	<0.001
RS			<0.001
Intermediate risk vs. low risk	10.01	1.82–54.99	0.008
High risk vs. low risk	192.42	26.87–1377.73	<0.001

*OR, Odds ratio; CI, Confidence interval; LVI, Lymph vascular invasion; PR, Progesterone receptor; RS, Recurrence score*.

When using the TAILORx cutoffs, there were 0.0% (0 out of 4), 13.7% (21 out of 153), and 67.5% (54 out of 80) patients receive chemotherapy recommendation in the Low-, Intermediate-, and High-risk RS groups (*P* < 0.001, Table S3). In multivariate analysis, age ≤50 years (OR = 2.94, 95% CI 1.23–7.02, *P* = 0.015), grade III tumor (OR = 47.45, 95% CI 3.14–716.88, *P* = 0.005), lymph vascular invasion (OR = 13.00, 95% CI 1.95–86.47, *P* = 0.008), PR <20% (OR = 5.18, 95% CI 2.00–13.41, *P* = 0.001), Ki-67 ≥14% (OR = 4.56, 95% CI 1.98–10.53, *P* < 0.001), and RS (*P* < 0.001) were still significantly associated with chemotherapy recommendation (Table S4). Patients with RS > 25 were more frequently to recommend for adjuvant chemotherapy when compared with RS ≤ 25 group with an OR = 19.15 (95% CI 8.05–45.54, *P* < 0.001).

### Chemotherapy Recommendation Change and Actual Application in T1bN0 Patients

**Figure 1:**
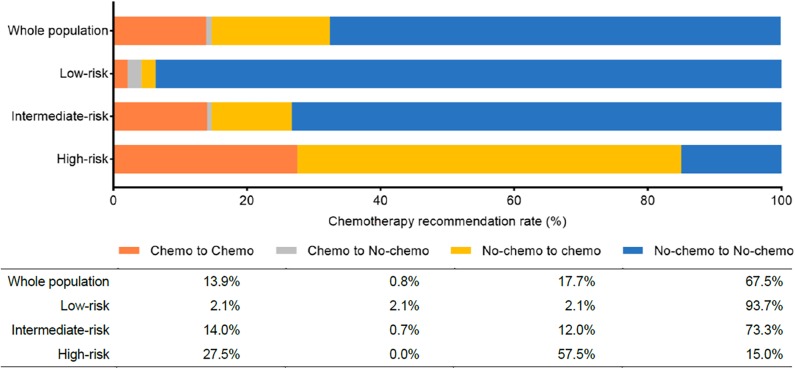
Chemotherapy recommendation change before and after the 21-gene RS testing. Chemo, chemotherapy; No-chemo, no chemotherapy.

There were 44 out of 237 patients (18.6%) received different treatment decisions before and after the 21-gene RS testing, among which 42 patients changed from no-chemotherapy to chemotherapy and only two patients (one had Low-risk RS and the other had Intermediate-risk RS) changed reversely. When stratified by different RS, treatment decision change occurred in 4.3% (2 out of 47), 12.7% (19 out of 150), and 57.5% (23 out of 40) patients in the Low-, Intermediate-, and High-risk RS groups, respectively (Table 5, Figure 1). When stratified by tumor grade, only one out of 55 (1.8%) with grade I tumor had treatment decision changed from chemotherapy to no-chemotherapy, while 31 (23.3%) patients with grade II tumor had recommendation shifts: all of which were from no-chemotherapy to chemotherapy. Regarding patients with grade III tumors, one patient changed from chemotherapy to no-chemotherapy, three patients changed reversely, and the decision change rate was 40.0% (Table S5).

**Table 5:** Chemotherapy recommendation before and after 21-gene RS testing in 237 patients with T1bN0 tumors.

**Post-RS**		**Pre-RS**		**Pre- to Post-changes**	**Actual application**		**Adherence to post-RS decision**
		**Chemo**	**No-chemo**		**Chemo**	**No-chemo**	
Whole	Chemo	33	42	44/237	66	9	226/237
	No-chemo	2	160	(18.6%)	2	160	(95.4%)
RS < 18	Chemo	1	1	2/47	2	0	47/47
	No-chemo	1	44	(4.3%)	0	45	(100.0%)
RS 18–30	Chemo	21	18	19/150	32	7	142/150
	No-chemo	1	110	(12.7%)	1	110	(94.7%)
RS > 30	Chemo	11	23	23/40	32	2	37/40
	No-chemo	0	6	(57.5%)	1	5	(92.5%)

*RS, Recurrence score; Chemo, Chemotherapy; No-chemo, No-chemotherapy*.

Patients' adherence to treatment decisions after the 21-gene RS testing was 95.4% (226 out of 237). Moreover, the treatment compliance rate was higher than 90% (range, 92.5–100.0%) in each subgroup when stratified by RS and tumor grade (Table 5, Table S5).

### Follow-Up and Disease Outcome

After a median follow-up period of 22.69 ± 16.17 months, only one patient with RS of 22 had disease recurrence. The patient was 36 years old, diagnosed with a 0.6 cm tumor in her left breast. The pathology showed it to be infiltrating ductal carcinoma with vascular invasion. Endocrine therapy alone had been used, and after 15 months, contralateral ductal carcinoma *in situ* was found.

## Discussion

In the current study, we included 237 HR+/HER2–, node-negative breast cancer patients with T1b tumors who underwent the 21-gene RS testing. We found that ER expression, PR expression, and Ki-67 index were independently related to RS distribution. All patients were discussed in two rounds of MDT for their adjuvant treatments. Chemotherapy recommendation was given to 31.6% of T1bN0 patients, which was more frequent in those with higher grade, lymph vascular invasion, Luminal B subtype, and Intermediate- or High-risk RS tumors. Adjuvant chemotherapy decision was changed in 18.6% of patients after physicians knowing the 21-gene RS results, which was mainly from non-chemotherapy to chemotherapy and mostly occurred to patients with Intermediate- to High-risk RS, indicating that physician-intended recommendation would be influenced by the 21-gene RS testing. Moreover, we found the adherence rate to the post-RS decision was higher than 90.0% in these T1bN0 patients, which partly due to the MDT and the 21-gene RS testing.

The popularity of breast screening and self-exam greatly contributed to the decrease in tumor size in the past few decades. Data from Surveillance, Epidemiology, and End Results (SEER) program showed that the proportion of small tumors had an increase of nearly 30% (7) from 1975 to 2012. This phenomenon merits concern and further investigation of the clinical feature and treatment pattern of small tumors. Several studies reported that tumor size is not an independent prognostic factor in T1a and T1b tumors (14–17). In another hand, distinct clinical outcomes were observed in a Korean cohort of small tumors according to molecular subtypes (18), among which HR+/HER2– breast cancer accounted for 56.6% with the best prognosis. For those patients, endocrine therapy could reduce the risk of recurrence, whereas the benefit of adjuvant chemotherapy should be weighed against the treatment-related risk. The predictive value of RS on chemotherapy benefit was universally acknowledged, especially when the result of TAILORx was published. Thus, for all breast tumor ≥0.5 cm, the NCCN guideline strongly recommends the 21-gene assay. But there was no specific data about the clinical significance of 21-gene RS in T1bN0 population. So, we performed the current study in the T1bN0 patients to describe the biological characteristics of small tumors manifested by the RS, as well as to figure out whether the adjuvant chemotherapy decision and actual administration would be influenced by the 21-gene testing in this group of patients.

Regarding the distribution of RS, our results showed that Low-, Intermediate-, High-risk RS accounts for 19.8, 63.3, and 16.9% in the T1bN0 cohort. The distribution observed by our previous study regardless of the tumor size was 26.1, 49.3, and 24.6% in three groups, respectively (19). We noticed that the proportion of Low- or High- risk RS decreased and more tumors were categorized as Intermediate-risk. We postulated that the small tumor size (≤ 1 cm) in the current study contributes to the difference, since small tumors may be associated with relatively better biological behavior (20, 21), as a reason for the lower proportion of High-risk. Meanwhile, patients with T1a tumors were excluded in this study, who may more likely to be genetically Low-risk. Pomponio et al. reported that the proportion of three RS groups account for 65.6, 29.9, and 4.5% in T1b tumors (22), and the NCDB data was 59.0, 33.4, 7.6%, respectively (23). The discordance between our results and theirs' may attribute to the genetic disparities between Chinese and western (24). Another possible reason may be due to the difference in enrollment criteria for the 21-gene RS testing, since we consecutively performed the assay on eligible patients, whereas other institutions may select patients by other clinical parameters.

Many pieces of research focused on clinicopathological factors associated with RS in order to find possible surrogates for the 21-gene testing, among which tumor grade and PR status were the most often discussed (20, 25). We noticed that in T1bN0 tumors, ER expression, PR expression, and Ki-67 index were significantly associated with the RS category. The proportion of patients with high-grade tumors was 2.1, 3.3, and 10.0% in Low-, Intermediate-, and High-risk RS groups, whereas we didn't observe the influence of tumor grade on RS in multivariate analysis, which may ascribe to the limitation of tumor size ≤1.0 cm. With respect to the IHC assessment of ER, PR, and Ki-67, we used 50% as a cutoff for ER expression and found that more than 90% of patients had high ER expression. The cutoff for PR status (20%) and Ki-67 index (14%) was according to St. Gallen 2013 expert panel. We suspected that selecting different cutoff points for IHC results may lead to discordance in results. Meanwhile, we had to admitted that the IHC-based classification may be more feasible but imperfect when comparing with the gene-based subtyping of intrinsic subtype (26, 27), and additional assays to clarify the biological diversity may also warrant investigation.

The chemotherapy usage was significantly associated with tumor grade, lymph vascular invasion, Luminal subtype, and RS in our study. Chemotherapy usage rate was 4.3, 26.0, and 85.0% in Low-, Intermediate-, and High-risk RS groups, which correspond approximately to the literature (23). Several studies and guidelines highlight the superiority of the 21-gene testing over routine clinicopathological parameters (28, 29). Meanwhile, the significance of tumor grade on chemotherapy usage, especially in tumors <1 cm, was also noted constantly (30, 31). Among all the independent factors in our study, High-risk RS had the highest OR for chemotherapy usage, followed by grade III tumors. This result could reflect the significant importance of these two factors in T1bN0 patients when the treatment decision was made. Furthermore, Ignatov et al. observed that in T1a/b breast cancer, the effect of systemic therapy on survival could only be seen in Luminal-B like tumors but not in Luminal-A like ones (30). Our physicians may take this into consideration when determining the therapeutic recommendation, so Luminal-B like patients were more likely to be asked for chemotherapy.

In the practice of clinic, the alteration in chemotherapy recommendation occurred to ~30% of cases after physicians knowing the RS (32, 33), and the application of the multi-gene testing was accompanied by the decrease in chemotherapy usage (34, 35). We focused on T1bN0 tumors and found that the decision change rate was 18.6%. Most of the changes (42 out of 44, 95.5%) were found in patients with Intermediate-risk (19 out of 150) or High-risk (23 out of 40) tumors, leading to an escalation in treatment pattern. Our results were consistent with the previous article, that adjuvant chemotherapy was given more frequently in T1bN0 patients who underwent the Oncotype DX (10.0 vs. 3.6%, *P* < 0.01) (21). The change in the opposite trend with real-world data may have two possible explanations. First, we performed two rounds of MDT for each patient and prospectively observed the change in chemotherapy decision, which is different from the study of Parsons et al. They draw the conclusion by performing multivariate analysis of NCDB data in which candidates for chemotherapy were determined by NCCN criteria (34). Another reason was that small-size tumors deserve less chemotherapy usage basically, in which Intermediate- to High-risk RS would be considered as a biomarker of a dire prognosis during decision-making procedure. In the current study, we did find that the 21-gene RS testing would influence the physician-intended recommendation for early breast cancer patients. However, the real benefit for chemotherapy change after 21-gene RS testing might be limited in T1bN0 population due to the relatively superior disease outcome of those patients. Moreover, the result calls for cautious interpretation since 62.4% of patients in our study were older than 50 years, for whom the benefit from chemotherapy was debatable even with intermediate-risk RS in the TAILORx study (6). However, Pomponio et al. founded that in T1bN0 patients, the performance of Oncotype DX may lengthen DFS by an average of 18.5 months (22), which can also illustrate the necessity of multi-gene testing in T1bN0 population. Furthermore, we noticed that for those T1bN0 patients with grade I tumors, who were not included in the TAILORx trial (11), the change due to multi-gene testing was only 1.8%. Thus, it may be reasonable to exempt those patients from the 21-gene testing, which can decrease healthcare costs at the same time. Furthermore, patients' compliance with the Post-RS decision was impressive in the present study, as high as 95.4%. The multi-gene assay may contribute to this since an adherence rate of 91% had also been reported by using the 70-gene signature (36). On the other hand, the two rounds of MDT in the decision-making process render the therapeutic schedule more reliable and acceptable.

Clinical outcomes of T1bN0 tumors were constantly excellent with 5-year DFS over 95.0% (8, 10, 16, 37). HR+ breast cancer was associated with ~20–30% lower risk of death when compared with other molecular subtypes (17) and had the best prognosis in small tumors (18, 38). Parise et al. found that 5-year Breast cancer-specific survival of HR+ T1b patients was 99.4% (39), which was concordant with the result published by the NCCN database (40). In our study, due to the small number of patients, only one case of contralateral DCIS was observed who received endocrine therapy alone.

Our study was designed to evaluate the role of 21-gene RS testing in T1bN0 patients which were outside of the TAILORx study, but there were several limitations. The retrospective design was the major weakness since the validation of the results may be negatively influenced by selection or information bias. We used multivariate analysis to narrow the confounding effect. Secondly, we used the IHC method for molecular subtyping instead of the intrinsic subtype identified by gene-based assay. It was because the latter one is not feasible for large-scale clinical applications, though maybe more valid to decipher the heterogeneity of breast cancer. Thirdly, the small number of patients together with the short follow-up period leads to few recurrent events to investigate the survival outcome difference, thus further evaluation is worthy of consideration.

## Conclusion

Our study included patients with HR+/HER2–, T1bN0 breast tumor and found that RS distribution was associated with ER expression, PR expression, and Ki-67 index, which was independently influenced adjuvant chemotherapy decision. The performance of the 21-gene RS testing would lead to an 18.6% change in therapeutic recommendation but rarely for low-grade tumors. Patient compliance to MDT suggestion was high after the 21-gene RS testing, which warrants further evaluation.

## Data Availability Statement

The raw data supporting the conclusions of this article will be made available by the authors, without undue reservation, to any qualified researcher.

## Ethics Statement

The studies involving human participants were reviewed and approved by Ethical Committees of Ruijin Hospital, Shanghai Jiao Tong University School of Medicine. Written informed consent for participation was not required for this study in accordance with the national legislation and the institutional requirements.

## Author Contributions

JY and XC contributed conception and design of the study. JW, OH, JH, ZL, WC, and YL organized the database. JY performed the statistical analysis and wrote the first draft of the manuscript. KS and XC contributed to manuscript revision and funding acquisition. All authors read and approved the submitted version.

## Conflict of Interest

The authors declare that the research was conducted in the absence of any commercial or financial relationships that could be construed as a potential conflict of interest.
