# Supplementary material for: Do 21-Gene Recurrence Score Influence Chemotherapy Decisions in T1bN0 Breast Cancer Patients?
Source: Front Oncol. 2020 May 12;10:708. doi: 10.3389/fonc.2020.00708 (PMC7236800; doi:10.3389/fonc.2020.00708)
Supplement: Supplementary file 1 [file Data_Sheet_1.doc]

Supplementary Material

**Table S1** Clinicopathologic characteristics of T1bN0 patients according to Recurrence Score (TAILORx standard)

|  | **Total**  **(n=237)** | **RS≤25**  **(n=80)** | **RS >25**  **(n=157)** | **P value** |
| --- | --- | --- | --- | --- |
| Age (years) |  |  |  | 0.768 |
| ≤50 | 89 (37.6%) | 60 (38.2%) | 29 (36.3%) |  |
| >50 | 148 (62.4%) | 97 (61.8%) | 51 (63.7%) |  |
| Comorbidity |  |  |  | 0.207 |
| No | 158 (66.7%) | 109 (69.4%) | 49 (61.3%) |  |
| Yes | 79 (33.3%) | 48 (30.6%) | 31 (38.8%) |  |
| Menstrual status |  |  |  | 0.049 |
| Pre-menopausal | 107 (45.1%) | 78 (49.7%) | 29 (36.3%) |  |
| Post-menopausal | 130 (54.9%) | 79 (50.3%) | 51 (63.7%) |  |
| Histologic type |  |  |  | 0.642 |
| IDC | 207 (87.3%) | 136 (86.6%) | 71 (88.8%) |  |
| Non-IDC | 30 (12.7%) | 21 (13.4%) | 9 (11.3%) |  |
| Tumor Grade |  |  |  | 0.051 |
| Grade I | 63 (26.6%) | 50 (31.8%) | 13 (16.3%) |  |
| Grade II | 133 (56.1%) | 81 (51.6%) | 52 (65.0%) |  |
| Grade III | 10 (4.2%) | 5 (3.2%) | 5 (6.3%) |  |
| Unknown | 31 (13.1%) | 21 (13.4%) | 10 (12.5%) |  |
| LVI |  |  |  | 0.978 |
| Yes | 9 (3.8%) | 6 (3.8%) | 3 (3.8%) |  |
| No | 228 (96.2%) | 151 (96.2%) | 77 (96.2%) |  |
| ER status |  |  |  | 1.000* |
| <50% | 7 (3.0%) | 5 (3.2%) | 2 (2.5%) |  |
| ≥50% | 230 (97.0%) | 152 (96.8%) | 78 (97.5%) |  |
| PR status |  |  |  | 0.003 |
| <20% | 48 (20.3%) | 134 (85.4%) | 55 (68.8%) |  |
| ≥20% | 189 (79.7%) | 23 (14.6%) | 25 (31.3%) |  |
| Ki-67 index |  |  |  | 0.006 |
| <14% | 153 (64.6%) | 111 (70.7%) | 42 (52.5%) |  |
| ≥14% | 84 (35.4%) | 46 (29.3%) | 38 (47.5%) |  |
| Luminal subtype |  |  |  | <0.001 |
| Luminal A-like | 118 (49.8%) | 94 (59.9%) | 24 (30.0%) |  |
| Luminal B-like | 119 (50.2%) | 63 (40.1%) | 56 (70.0%) |  |

Abbreviation: RS = Recurrence Score, IDC = invasive ductal carcinoma, LVI = Lymph vascular invasion, ER = Estrogen receptor, PR = Progesterone receptor.

*Fisher’s exact test

**Table S2** Multivariant analysis of characteristics associated with high risk RS (TAILORx standard, RS >25) in T1bN0 patients

|  | **OR** | **95% CI** | **p value** |
| --- | --- | --- | --- |
| Menstrual status (Pre vs. Post) | 1.94 | 1.02-3.68 | 0.043 |
| Tumor Grade |  |  | 0.244 |
| Grade II vs. Grade I | 0.48 | 0.23-1.02 | 0.058 |
| Grade III vs. Grade I | 0.36 | 0.79-1.65 | 0.189 |
| Unknown vs. Grade I | 0.70 | 0.25-1.97 | 0.503 |
| PR status (≤20% vs. >20%) | 0.97 | 0.33-2.86 | 0.954 |
| Ki-67 index (≥14% vs. <14%) | 1.25 | 0.40-3.96 | 0.703 |
| Luminal subtype (Luminal-B like vs. Luminal A like) | 3.81 | 1.06-13.71 | 0.041 |

Abbreviation: RS = Recurrence Score, PR = Progesterone receptor.

**Table S3** Factors associated with chemotherapy decision in T1bN0 patients

|  | **Chemo**  **(n = 75)** | **No-chemo**  **(n = 162)** | **P value** |
| --- | --- | --- | --- |
| Age (years) |  |  | 0.024 |
| ≤50 y | 36 (40.4%) | 53 (59.6%) |  |
| >50 y | 39 (26.4%) | 109 (73.6%) |  |
| Comorbidity |  |  | 0.236 |
| No | 54 (34.2%) | 104 (65.8%) |  |
| Yes | 21 (26.6%) | 58 (73.4%) |  |
| Menstrual status |  |  | 0.149 |
| Pre-menopausal | 39 (36.4%) | 68 (63.6%) |  |
| Post-menopausal | 36 (27.7%) | 94 (72.3%) |  |
| Histologic type |  |  | 0.142 |
| IDC | 69 (33.3%) | 138 (66.7%) |  |
| Non-IDC | 6 (20.0%) | 24 (80.0%) |  |
| Tumor Grade |  |  | <0.001 |
| Grade I | 9 (14.3%) | 54 (85.7%) |  |
| Grade II | 50 (37.6%) | 83 (62.4%) |  |
| Grade III | 9 (90.0%) | 1 (10.0%) |  |
| Unknown | 7 (22.6%) | 24 (77.4%) |  |
| LVI |  |  | 0.030* |
| Yes | 6 (66.7%) | 3 (33.3%) |  |
| No | 69 (30.3%) | 159 (69.7%) |  |
| ER status |  |  | 1.000* |
| <50% | 2 (2.7%) | 5 (3.1%) |  |
| ≥50% | 73 (97.3%) | 157(96.9%) |  |
| PR status |  |  | <0.001 |
| <20% | 26 (54.2%) | 22 (45.8%) |  |
| ≥20% | 49 (25.9%) | 140 (74.1%) |  |
| Ki-67 index |  |  | <0.001 |
| <14% | 28 (18.3%) | 125 (81.7%) |  |
| ≥14% | 47 (56.0%) | 37 (44.0%) |  |
| Luminal subtype |  |  | <0.001 |
| Luminal A-like | 13 (11.0%) | 105 (89.0%) |  |
| Luminal B-like | 62 (52.1%) | 57 (47.9%) |  |
| Recurrence Score |  |  | <0.001 |
| RS<11 | 0 (0.0%) | 4 (100.0%) |  |
| RS 11-25 | 21 (13.7%) | 132 (86.3%) |  |
| RS>25 | 54 (67.5%) | 26 (32.5%) |  |

Abbreviation: RS = Recurrence Score, IDC = invasive ductal carcinoma, LVI = Lymph vascular invasion, ER = Estrogen receptor, PR = Progesterone receptor.

*Fisher’s exact test

**Table S4** Multivariant analysis of characteristics associated with chemotherapy decision (RS with TAILORx standard)

|  | **OR** | **95% CI** | **p value** |
| --- | --- | --- | --- |
| Age (≤50y vs. >50y) | 2.94 | 1.23-7.02 | 0.015 |
| Tumor Grade |  |  | 0.021 |
| Grade II vs. Grade I | 1.98 | 0.73-5.37 | 0.181 |
| Grade III vs. Grade I | 47.45 | 3.14-716.88 | 0.005 |
| Unknown vs. Grade I | 0.75 | 0.17-3.38 | 0.705 |
| LVI (Positive vs. Negative) | 13.00 | 1.95-86.47 | 0.008 |
| PR status (<20% vs. ≥20%) | 5.18 | 2.00-13.41 | 0.001 |
| Ki-67 index (≥14% vs. <14%) | 4.56 | 1.98-10.53 | <0.001 |
| Luminal subtype (Luminal-B like vs. Luminal A like) | 1.73 | 0.30-9.89 | 0.539 |
| RS (RS >25 vs. RS≤25) | 19.15 | 8.05-45.54 | <0.001 |

Abbreviation: OR = Odds Ratio, CI = Confidence Interval, LVI = Lymph vascular invasion, PR = Progesterone receptor RS = Recurrence Score.

**Table S5** Chemotherapy recommendation before and after 21-gene RS testing in patients with T1bN0 tumors according to tumor grade

| Post-RS | | Pre-RS | | Pre- to Post- change | Actual application | | Adherence to Post-RS decision |
| --- | --- | --- | --- | --- | --- | --- | --- |
|  |  | Chemo | No-chemo |  | Chemo | No-chemo |  |
| Whole | Chemo | 33 | 42 | 44/237  (18.6%) | 66 | 9 | 226/237  (95.4%) |
|  | No-chemo | 2 | 160 |  | 2 | 160 |  |
| Grade I | Chemo | 1 | 0 | 1/55  (1.8%) | 8 | 1 | 62/63  (98.4%) |
|  | No-chemo | 1 | 53 |  | 0 | 54 |  |
| Grade II | Chemo | 19 | 31 | 31/133  (23.3%) | 42 | 8 | 125/133  (94.8%) |
|  | No-chemo | 0 | 83 |  | 0 | 83 |  |
| Grade III | Chemo | 6 | 3 | 4/10  (40.0%) | 9 | 0 | 10/10  (100.0%) |
|  | No-chemo | 1 | 0 |  | 0 | 1 |  |

Abbreviation: RS = Recurrence Score, Chemo = chemotherapy, No-chemo = No-chemotherapy.
